# Classification of symptom-side predominance in idiopathic Parkinson’s disease

**DOI:** 10.1038/npjparkd.2015.18

**Published:** 2015-10-29

**Authors:** Delia-Lisa Feis, Esther A Pelzer, Lars Timmermann, Marc Tittgemeyer

**Affiliations:** 1 Max Planck Institute for Metabolism Research, Translational NeuroCircuitry Group, Cologne, Germany; 2 Department of Neurology, University Hospital Cologne, Cologne, Germany

## Abstract

Asymmetry of symptom onset in Parkinson’s disease (PD) is strongly linked to differential diagnosis, progression of disease, and clinical manifestation, suggesting its importance in terms of specifying a therapeutic strategy for each individual patient. To scrutinize the predictive value of this consequential clinical phenomenon as a neuromarker supporting a personalized therapeutic approach, we modeled symptom-side predominance at disease onset based on brain morphology assessed with magnetic resonance (MR) images by utilizing machine learning classification. The integration of multimodal MR imaging data into a multivariate statistical model led to predict left- and right-sided symptom onset with an above-chance accuracy of 96%. By absolute numbers, all but one patient were correctly classified. Interestingly, mainly hippocampal morphology supports this prediction. Considering a different disease formation of this single outlier and the strikingly high classification, this approach proves a reliable predictive model for symptom-side diagnostics in PD. In brief, this work hints toward individualized disease-modifying therapies rather than symptom-alleviating treatments.

## Introduction

Parkinson’s disease (PD) is one of the most common neurodegenerative diseases resulting in motor as well as in non-motor dysfunctions. Despite its heterogeneous clinical manifestation and course, the asymmetry of motor symptoms at the time of diagnosis is a pathological hallmark indicating PD (i.e., idiopathic Parkinsonian Syndrome (PS)) that clinically helps to differentiate from atypical forms of PS.^1^ Besides this important role in the definition of PD, side of disease onset and progression of motor impairment were reported to be strongly linked:^1^ while a right-sided symptom onset is associated with a more favorable outcome in terms of cognitive impairment, a left-sided symptom onset appears to be associated with a better outcome regarding motor progression.^1–3^ Both clinical pathways certainly imply considerable difference not only in forecasting potential progression, but also the individual therapeutic approach. Although the origin of a unilateral symptom prevalence still remains enigmatic, this has sparked much interest in studying symptom-side predominance.^2^ While the clinical definition of a side predominance is not trivial with respect to the individual course of the disease, the determination at disease onset seems consistent.^2,3^

To date, numerous studies have employed multivariate decoding methods such as support vector machines to classify disease entities; rather infrequently these methods were applied to predict a treatment outcome or prognose disease progression at an individual subject level. With respect to PD, decoding methods have been used to delineate, e.g., essential tremor with rest tremor from tremor-dominant PD patients.^4^ Support vector classification involving data from quantitative magnetic resonance imaging (MRI) approaches, such as diffusion tensor imaging (DTI), has been recently applied to distinguish between PD patients and a group of patients with PD-mimicking conditions.^5^ Various white matter microstructural changes associated with PD^6^ thus reveal a significant hint at DTI as a valuable tool when investigating this pathology. In fact, decoding methods on morphological markers from various MRI methods have changed the culture of clinical cohort analysis to the extent that they putatively reveal underlying neurobiological mechanisms, hence supporting to move beyond descriptive phenomenological categorization.^7^

Following this rationale, one might argue that despite the lateralization of dopaminergic deficit, wide-spread structural differences reflecting the asymmetry of PD can be systematically found. Therefore, we introduced a multimodal approach to model symptom side at disease onset in brain morphology based only on different aspects of diffusion MR parameters and multi-kernel support vector classification.^8^ Using this approach, we deemed to identify specific morphological markers between disease forms unraveling the enigma of variety in clinical pattern of disease.

## Materials and methods

Methods and any associated references are available in the Supplementary Information of this paper.

## Results

**Figure 1:**
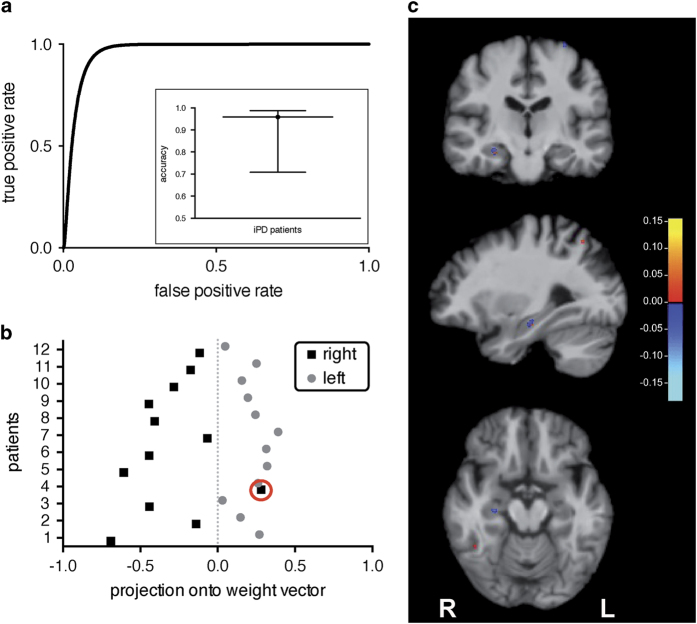
Classification performance with results mapped onto brain anatomy. (**a**) Receiver operating characteristic curve of the symptom-side predominance classification with an area under the curve of 0.9. The inset shows the classification accuracy with its credible interval ranging from 71 to 99%. (**b**) Best classification accuracy (96%) provided utilizing segregated brain gray and white matter segments. The red circle indicates the single misclassified patient. (**c**) Neuroanatomical findings of symptom-side predominance are superimposed onto a T_1_-weighted image of an individual study brain. The spatially contiguous patterns of discrimination reveal relatively higher cortical diffusivity (positive pattern vector; red color scale) or relatively lower cortical diffusivity (negative pattern vector; blue color scale) in left-sided patients. The largest predictive cluster is the posterior head of the right hippocampus (putatively CA4), which is illustrated in a coronal (first row), a sagittal (second row), and a horizontal slice (last row). The scale represents an arbitrary unit; L and R indicate the left and right hemispheres, respectively.

We used segregated brain gray (cortical aspect) and white (microarchitectural aspect) matter images of 24 male idiopathic PD patients to predict left- and right-sided symptom onset of motor signs. Remarkably, this framework yielded an above-chance accuracy of 96%. Classification performance is demonstrated by means of the receiver operating characteristics (ROC) curve with an area under the ROC curve of 0.9 (Figure 1a). The decision values that were provided by the classifier highlight the considerable separability of both groups (Figure 1b). In absolute terms, our classifier was able to correctly assign the symptom-side predominance in all but one individual (sensitivity of 100% and specificity of 92%). *Post hoc* inspection of the medical records of this one misclassified patient (red circle) revealed a suicide attempt with carbon monoxide poisoning, which differentiates him from all other patients.

When mapping the MRI relevant voxel that drive prediction results onto the neuroanatomical space (*cf.* Figure 1c), the largest discriminative cluster identified is the posterior head of the right hippocampus (putatively CA4).^9^ Since predominantly the pattern for explaining right-sided symptom onset (negative weight vector/blue color scale) occurred in this region, this finding might indicate relatively higher mean diffusivity in the right-sided symptom onset group as compared with the left-sided group.

## Discussion

We demonstrate a strikingly high prediction of symptom-side predominance in iPD patients that especially benefit from a novel multimodal whole-brain approach (due to the combination of white and gray matter segregations of different images). Our multi-kernel decoding algorithm discriminates body-side asymmetry with an accuracy of 96%, indicating excellent statistical performance. Apart from one outlier, this predictive model yields a remarkable precision of 100%. The exact determination of symptom-side prevalence at disease onset is of major interest since it has recently been linked to disease progression.^1^ Notably, *post hoc* mapping of most relevant regions revealed an aspect of the posterior head of the right hippocampus.^9^ One might speculate, that right-sided onset patients exhibit relatively more microstructural alterations in this area as compared with left-sided onset patients. This discriminative region is especially intriguing given the hippocampal role in spatio-temporal orientation and mnestic function.^9^ This also resonates with previous findings, where patients with a right-sided symptom onset were associated with a more favorable outcome regarding cognitive impairment, and a left-sided symptom onset appeared to be associated with a more favorable outcome in terms of motor progression.^1–3^ Here, our sole outlier attempted suicide by carbon monoxide poisoning 3 years prior to our study. As hippocampal damage is one of the typical pathological features of carbon monoxide poisoning,^10^ we argue that this is a reasonable explanation for his misclassification.

We explicitly chose the utility of diffusion-weighted brain imaging data here since these data are susceptible for the neurodegenerative aspects of PD. In particular, we used a combination of fractional anisotropy (segregated white matter), as well as mean diffusivity (segregated gray matter) images to investigate both the integrity of the fiber architecture and cortical differences at the same time (multimodal aspect). Especially the use of diffusion-weighted images enables the detection of subtle anomalies, as well as restricted diffusion in white and gray matter in patients suffering from carbon monoxide poisoning.^10^ Given that (i) our framework is based on segments of DTIs and (ii) our major classification pattern was identified in the hippocampus, even subtle changes in this one patients’ hippocampus could have misled the model to predict the wrong body side.

In conclusion, because of the excellent performance and group separability, we are convinced this proves a reliable predictive model for symptom-side diagnostics in iPD patients based solely on parameters from DTIs. We consider these results a major step in further predictive clinical models of PD incorporating the many clinical aspects that determine individual prediction of progression, as well as of a personalized sustainable therapeutic approach. We propose that the described asymmetry parameter should be incorporated as a covariate when striving for a comprehensive and particularly generalizable future model of this disease.
