# Supplementary material for: Classification of symptom-side predominance in idiopathic Parkinson’s disease
Source: NPJ Parkinsons Dis. 2015 Oct 29;1:15018–. doi: 10.1038/npjparkd.2015.18 (PMC5516555; doi:10.1038/npjparkd.2015.18)
Supplement: Supplementary Information [file npjparkd201518-s1.pdf]

SUPPLEMENTARY INFORMATION

Patients and methods

Patients. Given morphological gender differences in healthy subject [1,2] as well as in PD patients and the fact that men have a higher disease prevalence[3] we exclusively studied 24 right-handed male patients with idiopathic PD (iPD). The symptom-side assignment of left and right body-side was done according to Tomer et al.[4] by a neurologist with expertise in PD (E.A.P.). Our sample constituted of 12 left-sided and 12 right-sided symptom-onset patients, who did not differ in age, disease duration, their Levodopa equivalent daily dose or the degree of motor impairment off medication as measured by the unified Parkinson's disease rating scale-III (UPDRS III). For more information see Supplementary Table S1.

MRI acquisition parameters. Anatomical high-resolution T_1-weighted image were acquired using a 12-channel array head coil with a full-brain field of view (MDEFT3D: TR= 1930 ms, TI= 650ms, TE= 5.8 ms, 128 sagittal slices, resolution = 1x1x1.25mm^3, flip angle = 18°). The VBM8 toolbox was used to preprocess these images as described in Feis et al. [5]. Additionally, diffusion-weighted magnetic resonance (MR) imaging data were collected separately with a 32-channel array head coil from our sample using spin-echo echo-planar imaging (spin-echo-EPI: TR = 11200 ms, TE = 87 ms, 90 axial slices, resolution = 1.7 × 1.7 × 1.7 mm^3). Diffusion weighting was isotropically distributed along 60 directions (b-value 1000 s/mm^2). Seven images without diffusion weighting were acquired at the beginning and after each block of ten diffusion-weighted images, providing an anatomical reference for motion correction. The arithmetic mean across three consecutive scanning sessions was computed in order to increase the signal-to-noise ratio of the diffusion-weighted images. Mean diffusivity (MD) and fractional anisotropy (FA) images were estimated by fitting a diffusion tensor to the data within each voxel. Resulting images were co-registered to T_1-weighted images and nonlinearly normalized to an MNI template before being segmented into gray and white matter segments. To exploit information jointly encoded by different diffusion parameters and to ensure a whole-brain analysis, gray matter segments of the MD images and white matter segments of the FA images were submitted to the classifier.

Prediction. To avoid statistics on a group level basis but rather classify individual patients, a multivariate machine learning technique called support vector machine (SVM, as implemented by Chang and Li [6]) with a linear kernel was employed. The procedure used here to predict the symptom-side predominance of male PD patients at disease onset was previously described in [1,5]. In order to benefit from distinct parameter-specific physical apertures to the different tissue properties, we slightly modified our algorithm by employing a multi-kernel approach with an

equal weighting of the two diffusion images [7]. Generally, an SVM learns from labeled example training data to differentiate given groups. The major aim is to create a multimodal model based on MD (gray matter) and FA (white matter) imaging data that can accurately predict previously unseen patients having either left- or right-sided symptom onset (testing step; the data was not provided in the training phase). To this end, a decision function is learnt that discriminates the given training data to one of the two group labels. In this study, left-sided patients form the positive class; right-sided form the negative class.

Since both the MD and FA images consist of more voxels than iPD patients in our study, we preselected the most important voxels via feature selection method to ensure accurate prediction accuracy. In accordance to previous articles [1,5], a method called Fisher's criterion was applied to find the top 100 most relevant features [8]. Using a (nested) leave-one-patient-out cross-validation procedure on the training set, we automatically optimized the SVM regularization parameter [9]. Subsequently, we applied the optimal model to an independent set of test patients. Statistics such as sensitivity, specificity, F-measure, classification accuracy with its 95% credible interval and the area under the receiver operating characteristics (ROC) curve were calculated [5,10] to assess the performance of our multimodal classifier.

References

1 Feis, D.-L., Brodersen, K. H., von Cramon, D. Y., Luders, E. & Tittgemeyer, M. in *Neuroimage* Vol. 70 250-257 (2013).

2 Goldstein, J. M. *et al.* in *Cereb. Cortex* Vol. 11 490-497 (2001).

3 Solla, P. *et al.* in *J. Neurol. Sci.* Vol. 323 33-39 (2012).

4 Tomer, R., Levin, B. E. & Weiner, W. J. Side of onset of motor symptoms influences cognition in Parkinson's disease. *Ann Neurol.* **34**, 579-584 (1993).

5 Feis, D.-L. *et al.* in *NeuroImage: Clinical* Vol. 2 903-911 (2013).

6 Chang, C. in *Citeseer* (2001).

7 Zhang, D. *et al.* in *Neuroimage* Vol. 55 856-867 (2011).

8 Furey, T. S. *et al.* in *Bioinformatics* Vol. 16 906-914 (2000).

9 Lemm, S., Blankertz, B., Dickhaus, T. & Müller, K.-R. in *Neuroimage* Vol. 56 387-399 (2011).

10 Brodersen, K. H., Ong, C. S., Stephan, K. E. & Buhmann, J. M. in *2010 20th International Conference on Pattern Recognition (ICPR)* 3121-3124 (IEEE, 2010).

Table S1 Patient demographics.

	Symptom-side predominance [mean ± SD]		p-values of the Mann-Whitney U Test
	left	right	
age	61 ± 10	64 ± 10	0.45
disease duration	8 ± 5	7 ± 4	0.97
handedness	89 ± 15	78 ± 27	0.42
UPDRS III OFF medication	30 ± 8	27 ± 13	0.30
UPDRS III ON medication	16 ± 3	13 ± 7	0.14
Levadopa equivalent daily dose	768 ± 404	533 ± 260	0.21

Age and disease duration are given in years. The amount of Levadopa equivalent daily dose is indicated in milligram.
